# The diagnostic value of ADC histogram and direct ADC measurements for coexisting isocitrate dehydrogenase mutation and O6-methylguanine-DNA methyltransferase promoter methylation in glioma

**DOI:** 10.3389/fnins.2022.1099019

**Published:** 2023-01-11

**Authors:** Zhiyan Xie, Jixian Li, Yue Zhang, Ruizhi Zhou, Hua Zhang, Chongfeng Duan, Song Liu, Lei Niu, Jiping Zhao, Yingchao Liu, Shuangshuang Song, Xuejun Liu

**Affiliations:** ^1^Department of Radiology, The Affiliated Hospital of Qingdao University, Qingdao, China; ^2^Department of Neurosurgery, Shandong Provincial Hospital Affiliated to Shandong First Medical University, Jinan, China; ^3^Department of Nuclear Medicine, The Affiliated Hospital of Qingdao University, Qingdao, China

**Keywords:** ADC histogram, isocitrate dehydrogenase mutation, O6-methylguanine-DNA methyltransferase promoter methylation, glioma, diffusion weighted imaging

## Abstract

**Objectives:**

To non-invasively predict the coexistence of isocitrate dehydrogenase (IDH) mutation and O6-methylguanine-DNA methyltransferase (MGMT) promoter methylation in adult-type diffuse gliomas using apparent diffusion coefficient (ADC) histogram and direct ADC measurements and compare the diagnostic performances of the two methods.

**Materials and methods:**

A total of 118 patients with adult-type diffuse glioma who underwent preoperative brain magnetic resonance imaging (MRI) and diffusion weighted imaging (DWI) were included in this retrospective study. The patient group included 40 patients with coexisting IDH mutation and MGMT promoter methylation (IDHmut/MGMTmet) and 78 patients with other molecular status, including 32 patients with IDH wildtype and MGMT promoter methylation (IDHwt/MGMTmet), one patient with IDH mutation and unmethylated MGMT promoter (IDHmut/MGMTunmet), and 45 patients with IDH wildtype and unmethylated MGMT promoter (IDHwt/MGMTunmet). ADC histogram parameters of gliomas were extracted by delineating the region of interest (ROI) in solid components of tumors. The minimum and mean ADC of direct ADC measurements were calculated by placing three rounded or elliptic ROIs in solid components of gliomas. Receiver operating characteristic (ROC) curve analysis and the area under the curve (AUC) were used to evaluate the diagnostic performances of the two methods.

**Results:**

The 10th percentile, median, mean, root mean squared, 90th percentile, skewness, kurtosis, and minimum of ADC histogram analysis and minimum and mean ADC of direct measurements were significantly different between IDHmut/MGMTmet and the other glioma group (*P* < 0.001 to *P* = 0.003). In terms of single factors, 10th percentile of ADC histogram analysis had the best diagnostic efficiency (AUC = 0.860), followed by mean ADC obtained by direct measurements (AUC = 0.844). The logistic regression model combining ADC histogram parameters and direct measurements had the best diagnostic efficiency (AUC = 0.938), followed by the logistic regression model combining the ADC histogram parameters with statistically significant difference (AUC = 0.916) and the logistic regression model combining minimum ADC and mean ADC (AUC = 0.851).

**Conclusion:**

Both ADC histogram analysis and direct measurements have potential value in predicting the coexistence of IDHmut and MGMTmet in adult-type diffuse glioma. The diagnostic performance of ADC histogram analysis was better than that of direct ADC measurements. The combination of the two methods showed the best diagnostic performance.

## Introduction

The guidelines for the molecular diagnosis of gliomas have continued to change over recent years. The 2016 WHO classification of tumors of the central nervous system inserted molecular characteristics into the diagnostic criteria of gliomas, which had previously relied on histological diagnosis, and the 2021 edition emphasized the importance of classifying tumors by the type of molecular feature (Louis et al., 2016, 2021; McNamara et al., 2022). Isocitrate dehydrogenase mutations (IDHmut) occur in a high proportion of grade II and III gliomas and secondary glioblastomas and a low proportion of primary glioblastomas (Yan et al., 2009; Chen et al., 2017). The overall survival of patients with grade III glioma and glioblastoma harboring IDHmut was significantly longer than that of patients with IDH wildtype (IDHwt) (Yan et al., 2009).

O6-methylguanine-DNA methyltransferase (MGMT) repairs the DNA damage induced by temozolomide, and therefore higher levels of MGMT lead to temozolomide resistance (Chen et al., 2017; Molinaro et al., 2019). Methylation of the MGMT promoter (MGMTmet) decreases MGMT protein expression, thereby increasing sensitivity to temozolomide (Chen et al., 2017). Previous studies showed that patients with MGMTmet with grade II or III glioma or glioblastoma have a longer overall survival compared with those with unmethylated MGMT promoter (MGMTunmet) (Binabaj et al., 2018; Schaff et al., 2020; Haque et al., 2021, 2022). Notably, previous studies showed that patients with the coexistence of IDHmut and MGMTmet (IDHmut/MGMTmet) with low-grade glioma or glioblastoma had the longest survival, followed by those with IDHmut or MGMTmet alone, while glioma patients with IDHwt and MGMTunmet (IDHwt/MGMTunmet) had the shortest survival (Molenaar et al., 2014; Tanaka et al., 2015). The coexistence of IDHmut and MGMTmet thus indicates a better patient prognosis. Therefore, clarifying the status of IDH mutation and MGMT promoter methylation is critical to assess the prognosis of glioma patients. While genomic sequence analysis of surgical or biopsy specimens for IDH mutation status and MGMT promoter methylation status is accurate, this approach can be time consuming and is invasive. Furthermore, there is a risk that the specimens obtained by biopsy are too small to yield results. Therefore, a non-invasive method to predict the molecular status before surgery is ideal.

Magnetic resonance imaging (MRI) is a routine preoperative examination of gliomas. Diffusion-weighted imaging (DWI) is the most used MRI examination and provides important information on tumor proliferation by detecting the diffusion of water molecules in neoplastic tissues (Charles-Edwards and deSouza, 2006). Both direct apparent diffusion coefficient (ADC) measurements and ADC histogram analysis have been applied to predict the status of molecules, but the diagnostic performances vary widely. Several studies have used direct ADC values to predict IDH mutation or MGMT promoter methylation status of gliomas, and the area under the curve (AUC) varied from 0.686 to 0.870 (Xing et al., 2017, 2019, 2022; Cindil et al., 2022). Other studies have applied ADC histogram to predict the molecular status of gliomas. Lee et al. (2015) used ADC histogram parameters to predict IDH1 mutation of high-grade gliomas; however, the diagnostic value was limited (AUC 0.707). Direct ADC measurement is simple to performed, but only a few voxels are obtained. Histogram analysis is time-consuming, but it can capture subtle differences that are not visible to the naked eye. Therefore, it is meaningful and worthful to compare the diagnostic performances of the two methods, which may help researchers select a better method to maximize the value of DWI.

The goal of our study was to use ADC histogram analysis and direct ADC measurements to non-invasively predict the coexistence of IDHmut and MGMTmet in adult-type diffuse gliomas. We then compared the diagnostic performances of the two methods.

## Materials and methods

### Subjects

**FIGURE 1:**
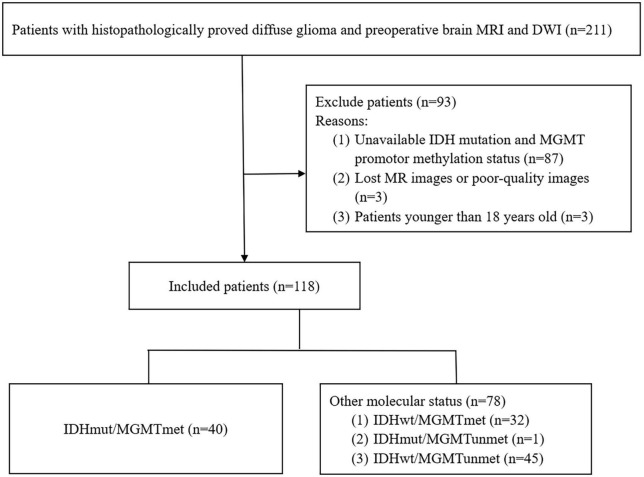
Flowchart of the collection of patients.

This retrospective study was approved by the medical ethics committee of the Affiliated Hospital of Qingdao University, and the requirement for informed consent was waived. The study included 211 patients with histopathologically proved diffuse glioma who underwent preoperative brain MRI and DWI between January 2017 and April 2022. None of the patients had received any brain treatment before the MRI scans. All patients underwent surgery within 2 weeks of the MRI scan. Exclusion criteria were as follows: (1) patients without information on IDH mutation and MGMT promoter methylation status; (2) patients with lost MR images or poor-quality images; and (3) patients younger than 18 years old. Finally, 118 patients (56 females and 62 males; mean age: 53.3 years; range: 21–75 years) with adult-type diffuse glioma were enrolled in the present study (Figure 1). IDH mutation and MGMT promoter methylation status were assessed by genomic sequence analysis using Sanger method and fluorescence quantitative PCR method.

### MR imaging protocols

Magnetic resonance imaging scans were performed using a 3.0 T or 1.5T MR scanner (Signa HDXt 3.0T, GE Healthcare, Milwaukee, WI, USA; Signa HDx 1.5T, GE Healthcare), using an eight-channel array coil. The MRI protocol included pre-contrast T1-weighted imaging (T1WI), T2-weighted imaging (T2WI), T2WI-fluid attenuated inversion recovery imaging (T2WI-FLAIR), and DWI. The repetition time (TR)/echo time (TE) of magnetic resonance sequences at the 3.0 T GE MR system were as follows: (1) T1WI: TR/TE = 2761/9 ms; (2) T2WI: TR/TE = 3040/99 ms; (3) T2WI-FLAIR: TR/TE = 8000/154 ms; and (4) DWI: TR/TE = 5100/76 ms. The TR/TE of magnetic resonance sequences at the 1.5 T GE MR system were as follows: (1) T1WI: TR/TE = 2612/20 ms; (2) T2WI: TR/TE = 3460/109 ms; (3) T2WI-FLAIR: TR/TE = 6004/126 ms; and (4) DWI: TR/TE = 4600/82 ms. DWI was performed with effective b values of 0 and 1000 s/mm^2^. ADC maps were reconstructed by DWI on the GE workstation.

### MR data processing

**FIGURE 2:**
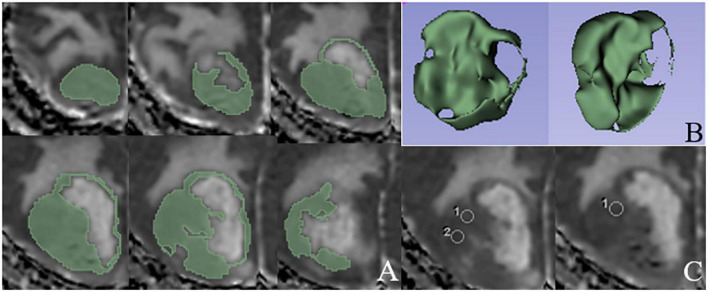
An example of ROIs delineated based on ADC histogram analysis and direct measurements, respectively. **(A)** ROIs manually delineated in solid components layer by layer on ADC map. **(B)** Three-dimensional stereogram generated by 3D Slicer software after the tumor segmentation. **(C)** Three rounded ROIs in solid components of the tumor based on the direct ADC measurements.

For ADC histogram analysis, tumor segmentation and feature extraction were implemented on 3D Slicer 4.11 software.^1^ Patient DICOM data were imported into 3D Slicer software by a radiologist with 3 years of neuroradiology experience. The radiologist was blinded to patient-related information and the histopathological and molecular results. ROIs were manually delineated in solid components of tumors layer by layer on ADC maps, and the necrotic, hemorrhagic, and cystic regions were avoided with reference to T1WI, T2WI, and T2WI-FLAIR (Figure 2). After the tumor was segmented, the pyradiomics module was applied to extracted ADC histogram parameters, including 10th percentile, mean, median, entropy, 90th percentile, interquartile range, minimum, kurtosis, maximum, skewness, mean absolute deviation, range, robust mean absolute deviation, uniformity, root mean squared, and variance.

The neuroradiologist measured minimum and mean ADC in solid components of tumors on the basis of the direct measurement method on AW workstation. Three rounded or elliptic ROIs were placed in solid components that were dark on ADC maps avoiding the necrotic, hemorrhagic, and cystic regions; the areas of ROIs ranged from 15–40 mm^2^ (Figure 2). The minimum and mean ADC of the three measurements were calculated.

After 2 months, the ROIs of all tumors on the basis of histogram analysis and direct measurement method were drawn again, and intraobserver agreement was assessed.

### Statistical analysis

The intraclass correlation coefficient (ICC) was used to evaluate intraobserver agreement; an ICC value more than 0.75 was considered as good consistency.

Statistical analyses were performed using the statistical package SPSS for Windows (Version 26.0, Chicago, IL, USA). Categorical variables such as gender and pathological grade were expressed as frequencies. Mean ADC, minimum ADC, kurtosis, skewness, and other continuous variables were expressed as mean and standard deviations (normal distribution) or median and quartiles (skewed distribution). The normal distribution of continuous data was assessed using Shapiro–Wilk test. The *t*-test (normal distribution) or Wilcoxon–Mann–Whitney test (heavily skewed distribution) was used to compare continuous variables between IDHmut/MGMTmet and gliomas with different mutation status, and the χ^2^ test was used to compare categorical variables. The variables that were statistically different between the two sets were included in the multivariate binary logistic regression analysis. Receiver operating characteristic (ROC) curve analysis and AUC were used to evaluate the diagnostic performances of the two methods. The cut-off value and the corresponding sensitivity and specificity were calculated by ROC curves. A *P*-value < 0.05 was considered statistically significant.

## Results

### ICC evaluation

The intraobserver agreement of ADC histogram parameters obtained from the two measurements was good (ICCs: 0.751–0.942). The minimum and mean ADC obtained from two direct measurements also showed good consistency (ICC: 0.934 and ICC: 0.945).

### Patient characteristics and pathological diagnosis of tumors

A total of 118 patients with adult-type diffuse glioma were enrolled in the present study. Among the 118 tumors, 40 tumors exhibited IDHmut/MGMTmet status. In the other 78 tumors, 32 tumors were IDHwt/MGMTmet, one tumor was IDHmut/MGMTunmet, and 45 tumors were IDHwt/MGMTunmet. Due to the lack of the results of EGFR gene amplification, +7/−10 chromosome copy number changes and CDKN2A/B homozygous deletion, the pathological diagnosis of tumors were carried out according to the 2016 WHO classification of tumors of CNS, including 5 diffuse astrocytoma, IDH-mutant (WHO II), 4 anaplastic astrocytoma, IDH-mutant (WHO III), 5 glioblastoma, IDH-mutant (WHO IV), 18 oligodendroglioma, IDH-mutant and 1p19q codeleted (WHO II), 9 anaplastic oligodendroglioma, IDH-mutant and 1p19q codeleted (WHO III), 4 diffuse astrocytoma, IDH-wildtype (WHO II), 8 anaplastic astrocytoma, IDH-wildtype (WHO III) and 65 glioblastoma, IDH-wildtype (WHO IV) (Louis et al., 2016, 2021).

There was no statistically significant difference in sex between the IDHmut/MGMTmet group and the other group (*P* = 0.995). Patients with IDHmut/MGMTmet gliomas were younger than patients with other molecular status (49.03 ± 11.77 vs. 55.53 ± 11.44 years, *P* = 0.005). The difference in the distribution of glioma grades between the IDHmut/MGMTmet group and the other group was statistically significant (*P* < 0.001). Lower grade gliomas (II + III) were the majority in IDHmut/MGMTmet gliomas, while glioblastoma (IV) was the majority in gliomas of other status. Comparison of sex, age, and pathological grade between the two groups is shown in Table 1.

**TABLE 1:** Comparison of sex, age, and pathological grade between IDHmut/MGMTmet and the other glioma group.

Demographics	IDHmut/MGMTmet, *N* = 40	Other molecular status (IDHwt/MGMTmet, *n* = 32; IDHmut/MGMTunmet, *n* = 1; IDHwt/MGMTunmet, *n* = 45), *N* = 78	*P*-value
Age (years)	49.03 ± 11.77	55.53 ± 11.44	0.005*
Sex [male, n (%)]	21 (52.5%)	41 (52.6%)	0.995
**Grade, n (%)**			
Lower grade (II + III)	36 (90.0%)	12 (15.4%)	<0.001*
Glioblastoma (IV)	4 (10.0%)	66 (84.6%)	

*P < 0.05.

### Diagnostic performance of the ADC histogram

**FIGURE 3:**
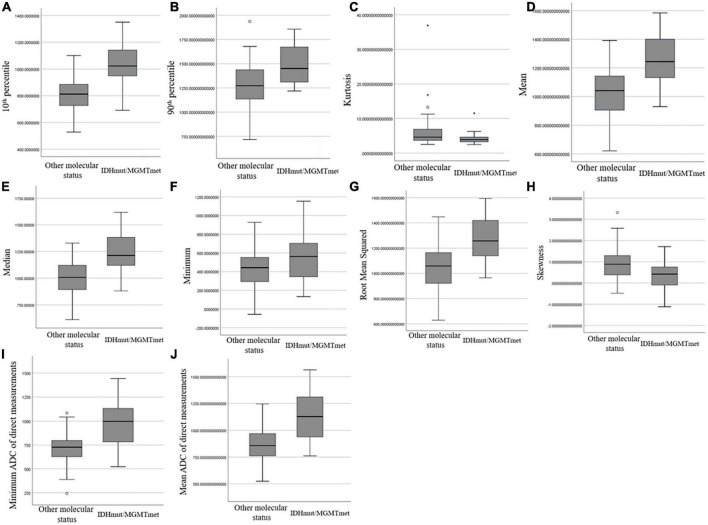
Box and whisker plot graphs showing comparison of ADC histogram parameters that were statistically different **(A–H)** and minimum ADC **(I)** and mean ADC **(J)** of direct measurements between IDHmut/MGMTmet and the other glioma group.

Comparison of ADC histogram parameters between IDHmut/MGMTmet and the other glioma group is shown in Table 2 and Figure 3. The 10th percentile, median, minimum, 90th percentile, mean, and root mean squared of IDHmut/MGMTmet gliomas were higher than those of the other glioma group, and the difference was statistically significant (*P* < 0.001 to *P* = 0.002). Kurtosis and skewness of IDHmut/MGMTmet gliomas were lower than those of the other group, and the difference was statistically significant (*P* = 0.003 and *P* < 0.001). The remaining ADC histogram parameters including maximum, uniformity, entropy, mean absolute deviation, range, interquartile range, variance, and robust mean absolute deviation showed no statistically significant differences between IDHmut/MGMTmet and gliomas with other molecular status.

**TABLE 2:** Comparison of ADC histogram parameters and minimum ADC and mean ADC between IDHmut/MGMTmet and the other glioma group.

Variable^#^	IDHmut/MGMTmet, *N* = 40	Other molecular status (IDHwt/MGMTmet, *n* = 32; IDHmut/MGMTunmet, *n* = 1; IDHwt/MGMTunmet, *n* = 45), *N* = 78	*P*-value
**ADC histogram parameters**			
10th percentile	1022.81 ± 152.53	803.55 ± 134.00	<0.001*
90th percentile	1451.00 (1309.00, 1679.75)	1273.50 (1135.00, 1439.28)	<0.001*
Entropy	4.89 (4.55, 5.14)	4.82 (4.57, 5.15)	0.695
Interquartile range	237.84 ± 79.60	232.37 ± 84.94	0.736
Kurtosis	3.89 (3.24, 4.65)	4.63 (3.66, 6.95)	0.003*
Maximum	2251.84 ± 508.33	2231.36 ± 532.18	0.841
Mean absolute deviation	147.18 ± 46.96	146.89 ± 51.14	0.976
Mean	1248.80 ± 165.37	1023.07 ± 171.02	<0.001*
Median	1238.63 ± 177.84	1002.16 ± 170.87	<0.001*
Minimum	556.75 ± 228.17	432.44 ± 186.38	0.002*
Range	1695.09 ± 664.53	1798.92 ± 604.99	0.395
Robust mean absolute deviation	99.81 ± 32.83	97.81 ± 35.11	0.766
Root mean squared	1264.64 ± 164.74	1042.41 ± 175.67	<0.001*
Skewness	0.30 ± 0.66	0.89 ± 0.68	<0.001*
Uniformity	0.040 (0.035, 0.051)	0.043 (0.036, 0.051)	0.481
Variance	36408.81 (20943.50, 53342.96)	34819.11 (22237.20, 57707.43)	0.887
**Direct ADC measurements**			
Minimum ADC	971.63 ± 226.79	719.32 ± 162.30	<0.001*
Mean ADC	1146.96 ± 218.15	870.15 ± 157.31	<0.001*

^#^Continuous variables with normal distribution were described as mean and standard deviations, and continuous variables with skewed distribution were described as median and quartiles. *P < 0.05.

The diagnostic performances of the ADC histogram parameters are shown in Table 3. The 10th percentile had the highest diagnostic efficiency (AUC: 0.860, 95% CI: 0.787–0.934), and the optimal cut-off value was 937.50 × 10^–6^ mm^2^/s with 80.0% sensitivity and 83.3% specificity. The AUC of median was 0.824 (95% CI: 0.748–0.900), followed by mean (AUC: 0.823, 95% CI: 0.748–0.899) and root mean squared (AUC: 0.818, 95% CI: 0.742–0.893). The AUC of 90th percentile, skewness, kurtosis, and minimum were 0.759, 0.726, 0.666, and 0.655, respectively.

**TABLE 3:** The diagnostic performances of ADC histogram parameters that were statistically different between IDHmut/MGMTmet and the other glioma group, minimum ADC and mean ADC of direct measurements and multivariate logistic regression models.

Variable	AUC	95% CI	Cut-off value	Sensitivity	Specificity
**ADC histogram parameters**					
10th percentile	0.860	0.787–0.934	937.50	80.0%	83.3%
90th percentile	0.759	0.673–0.846	1216.00	100.0%	42.3%
Mean	0.823	0.748–0.899	1108.79	85.0%	69.2%
Median	0.824	0.748–0.900	1085.50	85.0%	67.9%
Minimum	0.655	0.545–0.766	553.50	57.5%	75.6%
Root mean squared	0.818	0.742–0.893	1111.21	85.0%	67.9%
Kurtosis	0.666	0.567–0.765	5.07	87.5%	41.0%
Skewness	0.726	0.632–0.821	0.78	85.0%	59.0%
**Direct ADC measurements**					
Minimum ADC	0.810	0.721–0.899	945.50	65.0%	91.0%
Mean ADC	0.844	0.770–0.918	1073.17	67.5%	89.7%
**Multivariate logistic regression models**					
ADC histogram parameters	0.916	0.868–0.964		82.5%	85.9%
Direct ADC measurements	0.851	0.780–0.921		67.5%	88.5%
ADC histogram parameters + direct ADC measurements	0.938	0.896–0.980		87.5%	87.2%

AUC, area under the ROC curve; CI, confidence interval.

### Diagnostic performance of direct ADC measurements

Comparison of minimum ADC and mean ADC between IDHmut/MGMTmet and the other group is shown in Table 2 and Figure 3. The minimum ADC and mean ADC of IDHmut/MGMTmet gliomas were higher than those of other gliomas, and the difference was statistically significant (*P* < 0.001).

The diagnostic performances of minimum ADC and mean ADC are shown in Table 3. The AUC of mean ADC was 0.844 (95% CI: 0.770–0.918), and the optimal cut-off value was 1073.17 × 10^–6^ mm^2^/s with 67.5% sensitivity and 89.7% specificity. The AUC of minimum ADC was 0.810 (95% CI: 0.721–0.899), and the optimal cut-off value was 945.50 × 10^–6^ mm^2^/s with 65.0% sensitivity and 91.0% specificity.

### The diagnostic performance of the combination of the two methods

**FIGURE 4:**
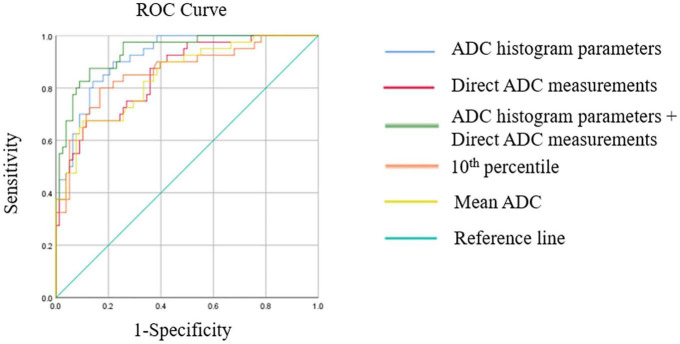
This figure shows receiver operating characteristics (ROC) curves of multivariate logistic regression models, the best ADC histogram parameter, and the best parameter of direct ADC measurements.

The diagnostic performances of multivariate logistic regression models are shown in Table 3 and Figure 4. The logistic regression model combining ADC histogram parameters and direct measurements had the best diagnostic efficiency (AUC: 0.938, CI: 0.896–0.980), followed by the logistic regression model combining the ADC histogram parameters with statistically significant difference (AUC: 0.916, CI: 0.868–0.964), and the logistic regression model combining minimum ADC and mean ADC (AUC: 0.851, CI: 0.780–0.921).

## Discussion

In this study, we demonstrated the value of DWI in predicting the coexistence of IDHmut and MGMTmet in adult-type diffuse glioma. Both ADC histogram and direct ADC values had good diagnostic performance, and the combination of the two methods had the best predictive value. Previous studies showed that the coexistence of IDHmut and MGMTmet significantly prolonged the overall survival of glioblastoma patients who received temozolomide and radiation therapy (Yang et al., 2015; Li et al., 2016), and IDH mutation and MGMT promoter methylation were independent predictive factors for pseudoprogression disease (Li et al., 2016). Additionally, Tanaka et al. (2015) reported that combined IDH1 mutation and MGMT promoter methylation was associated with a better prognosis in low-grade glioma. Therefore, the status of IDH mutation and MGMT promoter methylation are important prognostic factors for glioma. A previous study used mean relative ADC to differentiate IDH wild-type and IDH-mutant gliomas with an AUC of 0.790 (Wu et al., 2018). In the current study, the combination of ADC histogram and direct ADC measurements showed the highest value of DWI (AUC = 0.938), which contributed to predicting the prognosis of glioma patients.

In our study, the diagnostic performance of ADC histogram was better than direct ADC measurement in predicting the coexistence of IDHmut and MGMTmet in gliomas. Several studies have compared the diagnostic performance of ADC histogram analysis with direct measurements in tumor grading or differentiating benign from malignant tumors. Han et al. (2017) found that the diagnostic performance of whole-volume histogram analysis was not better than single-slice methods in glioma grading. Another study reported that whole-lesion ADC histogram analysis and single-slice ADC measurement had a similar diagnostic performance in differentiating benign and malignant soft tissue tumors (Ozturk et al., 2021). The reason why our results differed from those of previous studies may be that we selected variables with statistically significant differences to establish the multivariate logistic regression models. The results indicated that the AUC of the logistic regression model combining ADC histogram parameters was higher than that of the model combining parameters obtained by direct measurements.

Apparent diffusion coefficient histogram analysis uses descriptive parameters to characterize and compare distributions of ADC values in a quantitative manner (Just, 2014). In the current study, the ADC histogram parameter of 10th percentile, mean, median, 90th percentile, and minimum described distributions of ADC values of gliomas and the ADC values of IDHmut/MGMTmet gliomas were higher than those of other gliomas. Since lower cellular density led to higher ADC values, we concluded that the cellular density in IDHmut/MGMTmet gliomas was lower than that of other gliomas. The ADC histogram parameter of maximum of IDHmut/MGMTmet gliomas was not statistically significantly different from that of the other group. The reason may be that small cystic and necrotic areas were manually delineated into the ROIs, which was inevitable, even with conscious efforts to avoid cystic and necrotic areas.

Skewness of ADC histogram represents a measure of asymmetrical distribution of ADC value, and an ADC histogram is generally considered positively skewed if it has an elongated tail on the right side of the mean (Just, 2014). In our study, the skewness value of gliomas with other molecular status was positive and higher than that of IDHmut/MGMTmet gliomas; the difference was statistically significant. Therefore, the skewness of gliomas with other molecular status was more positive than IDHmut/MGMTmet gliomas and the shape of ADC histogram of the former was more asymmetric than the latter. A previous study reported that the change in ADC histogram skewness may be associated with early treatment response to anti-angiogenic therapy in patients with recurrent high-grade glioma (Nowosielski et al., 2011). Our results showed that skewness was predictive of IDHmut/MGMTmet in gliomas.

Kurtosis of ADC histogram represents the peakedness of the distribution of ADC value. In our study, kurtosis was significantly different between IDHmut/MGMTmet and the other group, but its diagnostic value was limited. Root mean square refers to the standard deviation of ADC values of all voxels in the ROI. Our results showed that root mean square of IDHmut/MGMTmet gliomas was higher than that of the other group, and the root mean square had good diagnostic value.

Patients with IDHmut/MGMTmet gliomas were younger than patients with other molecular status in the current study, which was consistent with a previous study (Zhang et al., 2021). In addition, lower grade gliomas were the majority in IDHmut/MGMTmet gliomas, while glioblastoma was the majority in gliomas with other molecular status. This result was consistent with a previous study that reported that a high percentage of lower grade gliomas harbors mutations in IDH1 and IDH2 (Cohen et al., 2013).

Furthermore, the data were collected from MR scanners different field strength (1.5T and 3.0T). However, we compared age, sex, pathological grade, ADC histogram parameters and direct ADC values between 1.5T and 3.0T scanner group. The results were provided in Supplementary Tables 1, 2. All parameters with statistically difference between IDHmut/MGMTmet and the other glioma group showed no statistically difference between 1.5T and 3.0T scanner group. We concluded that scanners with different field strengths did not affect the results of the current study. In addition, a previous study reported that ADC is a field strength-independent parameter (Chawla et al., 2009). Another study scanned submandibular glands of three healthy volunteers at both 1.5 and 3.0 T scanners, and there was no statistical difference between ADC values measured on 1.5 and 3.0 T scanners (Kim et al., 2009).

This study has several limitations. First, it was a retrospective study with possible biases in patient selection. Second, the sample size of this single-center study was relatively small, and multicenter studies may be needed to obtain a larger sample size for future analysis. Third, due to the lack of the results of EGFR gene amplification, +7/−10 chromosome copy number changes and CDKN2A/B homozygous deletion (Louis et al., 2021), the pathology of tumors was diagnosed according to the 2016 WHO classification of tumors of CNS. However, gliomas were grouped according to the status of IDH mutation and MGMT promoter methylation in this study, and the results of WHO grade of tumors did not affect the main results of this study.

## Conclusion

Both ADC histogram analysis and direct measurements have potential value in predicting the coexistence of IDHmut and MGMTmet in adult-type diffuse gliomas. The diagnostic performance of ADC histogram analysis was better than that of direct ADC measurements. Furthermore, the combination of the two methods showed the best diagnostic performance.

## Data availability statement

The raw data supporting the conclusions of this article will be made available by the authors, without undue reservation.

## Ethics statement

The studies involving human participants were reviewed and approved by the Medical Ethics Committee of the Affiliated Hospital of Qingdao University. Written informed consent for participation was not required for this study in accordance with the national legislation and the institutional requirements.

## Author contributions

ZX and XL designed the idea and framework of the manuscript. CD, SL, LN, and JZ collected the data. SS, YZ, RZ, and HZ conducted a statistical analysis of the data. ZX and JL wrote the manuscript. YL, SS, and XL put forward revisions. All authors contributed to the article and approved the final version of the manuscript.

## Footnotes

1 Available at https://www.slicer.org.
