# Supplementary material for: The diagnostic value of ADC histogram and direct ADC measurements for coexisting isocitrate dehydrogenase mutation and O6-methylguanine-DNA methyltransferase promoter methylation in glioma
Source: Front Neurosci. 2023 Jan 11;16:1099019. doi: 10.3389/fnins.2022.1099019 (PMC9875074; doi:10.3389/fnins.2022.1099019)
Supplement: Supplementary file 1 [file Table_1.DOCX]

Comparison of age, sex and pathological grade between 1.5T and 3T scanner group is shown in **Supplementary Table 1**. There was no statistically significant difference in age, sex and pathological grade between the two groups (*P*=0.278, 0.344 and 0.168).

Comparison of ADC histogram parameters and direct ADC values between 1.5T and 3T scanner group is shown in **Supplementary Table 2**. Minimum ADC and mean ADC obtained by direct measurements as well as the ADC histogram parameters including 10^th^ percentile, 90^th^ percentile, kurtosis, maximum, mean, median, minimum, root mean squared and skewness showed no statistically significant differences between the two groups.

**Supplementary Table 1** Comparison of age, sex and pathological grade between 3T and 1.5T scanner group.

| Demographics | 3T scanner, N=88 | 1.5T scanner, N=30 | *P* value |
| --- | --- | --- | --- |
| Age (years) | 52.63±11.54 | 55.37±12.92 | 0.278 |
| Sex (male, n (%)) | 44(50.0%) | 18(60.0%) | 0.344 |
| Grade, n (%) |  |  |  |
| Lower grade (II+III) | 39(44.3%) | 9(30.0%) | 0.168 |
| Glioblastoma (IV) | 49(55.7%) | 21(70.0%) |  |

**Supplementary Table 2** Comparison of ADC histogram parameters and minimum ADC and mean ADC between 3T and 1.5T scanner group.

| Variable^#^ | 3T scanner, N=88 | 1.5T scanner, N=30 | *P* value |
| --- | --- | --- | --- |
| ADC histogram parameters |  |  |  |
| 10^th^ percentile | 886.33±184.52 | 853.06±140.71 | 0.308 |
| 90^th^ percentile | 1333.80±255.19 | 1366.58±212.34 | 0.528 |
| Entropy | 4.78(4.55, 5.11) | 5.01(4.80, 5.31) | 0.024^*^ |
| Interquartile range | 218.50(180.75, 259.25) | 253.00(216.25, 319.00) | 0.026^*^ |
| Kurtosis | 4.34(3.36, 5.52) | 4.45(3.74, 5.90) | 0.415 |
| Maximum | 2184.70±536.08 | 2395.52±451.07 | 0.056 |
| Mean absolute deviation | 141.65±49.17 | 162.66±48.11 | 0.044^*^ |
| Mean | 1101.12±211.68 | 1095.10±162.59 | 0.887 |
| Median | 1085.97±219.44 | 1071.63±162.53 | 0.743 |
| Minimum | 491.82±208.07 | 424.00±207.16 | 0.125 |
| Range | 1692.89±641.03 | 1971.52±531.85 | 0.034^*^ |
| Robust mean absolute deviation | 93.41(77.13, 110.17) | 107.11(91.35, 134.40) | 0.022^*^ |
| Root mean squared | 1118.12±213.06 | 1116.64±165.17 | 0.972 |
| Skewness | 0.63±0.78 | 0.87±0.50 | 0.060 |
| Uniformity | 0.044(0.037, 0.052) | 0.039(0.030, 0.044) | 0.030^*^ |
| Variance | 32616.09(21196.87, 53259.01) | 47858.58(31814.67, 63884.33) | 0.029^*^ |
| Direct ADC measurements |  |  |  |
| Minimum ADC | 774.50(633.00, 998.25) | 748.00(665.25, 839.75) | 0.330 |
| Mean ADC | 927.17(790.08, 1129.67) | 910.50(827.92, 983.83) | 0.451 |

^#^: Continuous variables with normal distribution were described as mean and standard deviations, and continuous variables with skewed distribution were described as median and quartiles.

^*^: *P*<0.05
